# Promoting Social Engagement With a Multi-Role Dancing Robot for In-Home Autism Care

**DOI:** 10.3389/frobt.2022.880691

**Published:** 2022-06-20

**Authors:** Hifza Javed, Chung Hyuk Park

**Affiliations:** Department of Biomedical Engineering, School of Engineering and Applied Science, George Washington University, Washington, DC, United States

**Keywords:** socially assistive robot (SAR), robot dancing, in-home ASD care, social engagement, longitudinal study

## Abstract

This work describes the design of real-time dance-based interaction with a humanoid robot, where the robot seeks to promote physical activity in children by taking on multiple roles as a dance partner. It acts as a leader by initiating dances but can also act as a follower by mimicking a child’s dance movements. Dances in the leader role are produced by a sequence-to-sequence (S2S) Long Short-Term Memory (LSTM) network trained on children’s music videos taken from YouTube. On the other hand, a music orchestration platform is implemented to generate background music in the follower mode as the robot mimics the child’s poses. In doing so, we also incorporated the largely unexplored paradigm of learning-by-teaching by including multiple robot roles that allow the child to both learn from and teach to the robot. Our work is among the first to implement a largely autonomous, real-time full-body dance interaction with a bipedal humanoid robot that also explores the impact of the robot roles on child engagement. Importantly, we also incorporated in our design formal constructs taken from autism therapy, such as the least-to-most prompting hierarchy, reinforcements for positive behaviors, and a time delay to make behavioral observations. We implemented a multimodal child engagement model that encompasses both affective engagement (displayed through eye gaze focus and facial expressions) as well as task engagement (determined by the level of physical activity) to determine child engagement states. We then conducted a virtual exploratory user study to evaluate the impact of mixed robot roles on user engagement and found no statistically significant difference in the children’s engagement in single-role and multiple-role interactions. While the children were observed to respond positively to both robot behaviors, they preferred the music-driven leader role over the movement-driven follower role, a result that can partly be attributed to the virtual nature of the study. Our findings support the utility of such a platform in practicing physical activity but indicate that further research is necessary to fully explore the impact of each robot role.

## 1 Introduction

In recent years, the field of Human-Robot Interaction (HRI) has seen tremendous advancements in understanding how to design effective interactions between humans and robots. One application of HRI is in the use of Socially Assistive Robots (SARs) to design autism interventions, offering opportunities for a positive impact on children’s emotional well-being, social relationships, mental health, and general skill development. Although HRI research has demonstrated that robot-assisted interventions can be very impactful in improving the quality of life of autistic children, designing truly effective autism interventions remains an open question, with yet more work to be done to develop a deeper understanding of the needs of the target population, accurate modeling of engagement and affect in humans, the objective measures required to monitor the complex processes of child learning and long-term behavioral changes, and the different ways in which we can fully leverage the benefits offered by SARs.

Physical activity has been found to improve psychological wellbeing leading to positive self-esteem and happiness in autistic children. However, the impairments in social communication and interaction that are characteristic of autism can influence the play activities of children, compelling them to resort to solitary, passive play (Memari et al., 2015; Holmes and Willoughby, 2005). Structured physical activity programs have also been found to positively influence social interaction and communication skills of autistic children (Pan, 2008; Zhao and Chen, 2018). These findings corroborate those from studies investigating the impact of music and dance therapies for autism, reinstating the positive relationship between physical activity and social skills (Berlandy, 2019; Thompson et al., 2014; Eren, 2015).

Music and dance also play integral roles in encouraging children to develop spontaneous self-expression, communication, and interaction by offering emotional and motivational media that can often be easier for children to assimilate. While these methods are popular in autism therapy, the use of SARs in implementing these techniques remains limited in the existing body of research. This is not surprising given the challenges of real-time motion synthesis and full-body motion realization with a humanoid robot.

In our work, we explore the use of music and dance therapy methods through a robot-assisted dance interaction framework that is aimed at promoting physical activity and encouraging social engagement in children and seek to explore different paradigms of robot behaviors within a dance interaction to offer diverse opportunities for engagement and physical activity.

In addition, the work presented here is also motivated by the development of in-home solutions for autism care. Providing care for an autistic child is a demanding and challenging role that may pose numerous stressors to the caregivers and can impact the family at large. The social stigma associated with certain symptomatic behaviors in autism can lead to severe mental health challenges for the child, as well as their family. Considering this risk, it becomes crucial for the child to receive therapy promptly and adequately to minimize these potential dangers. The financial burden of therapy is another aspect to consider when assessing the impact on families. Even when families can afford therapy services, obstacles can often take the form of insurance restrictions and limited access to trained professionals. It is not uncommon for children to experience prolonged waiting periods to receive diagnostic evaluation and therapy. Therefore, this works also aims to bridge the gap between the demand for and accessibility of autism care with in-home solutions and deliver effective music- and dance-based interactions through SARs.

### 1.1 Related Work

***Dance-Based cHRI Studies*** Several HRI studies in the past have explored the efficacy of incorporating dance interactions in their designs for applications ranging from autism interventions to entertainment, human-robot trust assessment, and dance tutoring. Pepper, a semi-humanoid robot, was used to design a dance interaction for school-going children (Venture et al., 2017), targeting an assessment of the children’s perception of the robot. Another semi-humanoid robot with a mobile base, called Maggie, was used to engage a user in partner dances (among other interaction scenarios) (Salichs et al., 2006). Nao, a bipedal humanoid robot platform, was programmed to perform multiple dances targeting improvements in physical activity, whereby the children were tasked to imitate the robot dance steps in real-time (Ros et al., 2011). Nao was also used in another study (Barnes et al., 2020) specifically targeting a dance-based autism interaction for children, in which a Dance Freeze game was implemented wherein the robot and the child both dance when the music plays and “freeze” when the music is abruptly paused.

Within the animaloid form factor, dance interactions were designed using Pleo (Curtis et al., 2011), Aibo (Angulo et al., 2011), and Keepon (Michalowski et al., 2007). The interaction design for Pleo specifically targeted therapy, though it was not formally evaluated with a user study. For Aibo, some basic dance steps were manually designed, which were then randomly combined to form multiple dance sequences. Keepon was used in a generalized dance interaction with children where its movements were controlled by a rhythm-based software.

Based on this review of related past work, several gaps can be identified including: 1) a lack of automation resulting in heavy dependence on human controller input to create real-time responsiveness and interactivity in a human-robot interaction, 2) a small library of robot dances with a general lack of variety as a result of manual choreography design, 3) no or limited ability of the robot dynamically generate relevant behavioral responses to changes in user behavior or the environment, 4) limited use of bipedal humanoid robots for executing dynamically generated, full-body dances, and 5) a lack of autism-specific interaction scenarios with deliberate incorporation of the principles and constructs of formal therapy.

These factors were used to inform the dance-based interaction design presented in this work, which addresses all the problem areas identified above in order to develop a more effective and socially interactive dance interaction for autistic children.

***Robot Roles in cHRI Research*** Robot-assisted interventions are typically designed to target a variety of symptoms that are commonly experienced by autistic individuals. However, most child robot interaction (cHRI) interaction designs use the robot in the roles of a teacher, instructor, demonstrator, behavior model, etc., where the primary use of the robot is to teach a skill or behavior without taking any feedback from the user that may impact its own behavior. In this sense, it is a unidirectional interaction since the robot mainly teaches and the child mainly learns. There are not many available interaction scenarios in the literature where the child takes control of the interaction while the robot learns from them.

For example, in an attempt to teach social skills to children, the robot may be used as a social actor to demonstrate the skill, thereafter, prompting the child to replicate that behavior (Pangrazi et al., 2003; Javed et al., 2019; Desideri et al., 2017; David et al., 2020). The robot may also be used to discourage negative behaviors in a child, in which case it gives negative feedback to the child upon detecting the said behavior (Robins et al., 2012). In both cases, the emphasis is on changing the child’s behavior by teaching the appropriate skill or behavior. It is uncommon for the robot behavior to be controlled by the child such that the child may teach appropriate behavior to the robot, and in the process of teaching, also learn the behavior themselves.

A large variety of other intervention targets can also be found in the literature, including (but not exhaustively) improvements in tolerance to tactile contact (Sandygulova et al., 2019), emotion interpretation (Sandygulova et al., 2019), object identification skills (such as vehicles and animals) (Sandygulova et al., 2019; Desideri et al., 2017), understanding the social context from storytelling (Elsabbagh et al., 2012), vocabulary building and other learning goals (Desideri et al., 2017), teaching everyday activities (such as personal hygiene and eating food, etc.) (Syrdal et al., 2020), and improving problem-solving skills through games (Clabaugh et al., 2019). The common theme in all these scenarios is the use of the robot primarily in the role of a teacher. A notable exception, however, is the application of robots as tutors for children where they may act as a tutor, a tutee, or both (Ros et al., 2016; Howard et al., 2017). Findings from these studies support the use of the robots in mixed roles in order to fully leverage their capacity to help children achieve their learning goals.

We used the lack of bidirectional learning scenarios in cHRI for autism-specific interactions to inform the work presented here, which explores the paradigm of learning-by-teaching, such that the robot can both lead and follow the child as their dance partner.

## 2 Materials and Methods

In our robot-assisted dance interaction design, the robot gains social awareness through multimodal behavioral sensing and acts with a high level of autonomy to engage a child in a dance activity. The process involves the four main stages: 1) sensing the behavioral cues through facial expression and pose capture, 2) interpreting the sensor data to understand the user engagement state (which is composed of an emotional state, an attentional state, and a physical activity level), 3) utilizing the user state information to decide the next best action for the robot to perform, and 4) executing the selected action.

**FIGURE 1:**
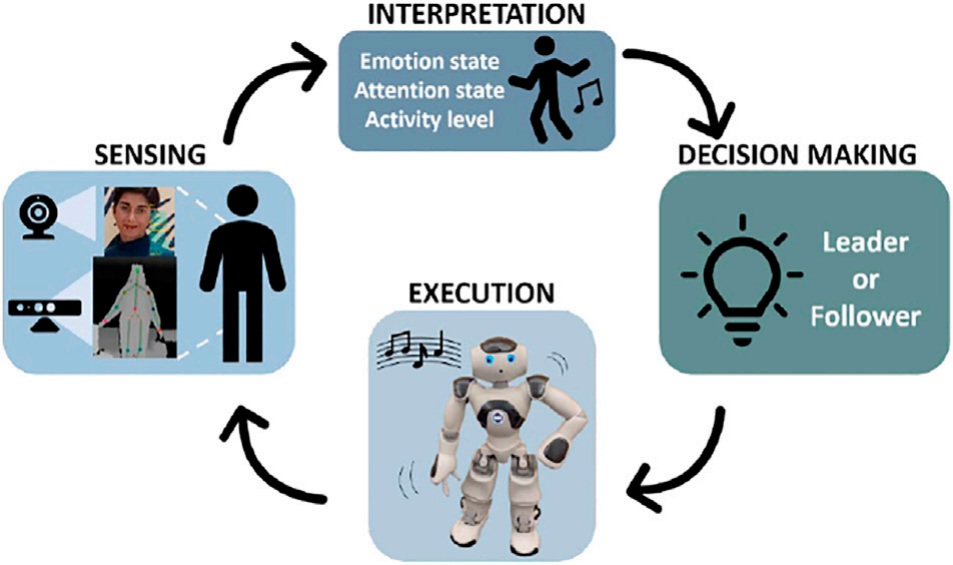
An overview of the control architecture for our largely autonomous child-robot dance interaction. Our system implements a cycle where it senses behavioral cues from a user, interprets these to determine user engagement, decides a suitable role for the robot to assume next, and then executes the selected robot action (Written informed consent was obtained from the individual(s) AND/OR minor(s)' legal guardian.).

This cycle is repeated until the interaction ends and is illustrated in Figure 1. The robot may act as either a leader or a follower-a decision that is made by a reinforcement learning-based module that learns from the social cues to decide the best course of action for the robot. This decision-making process is not discussed in this paper since its focus is on the evaluation of the two robot roles.

**FIGURE 2:**
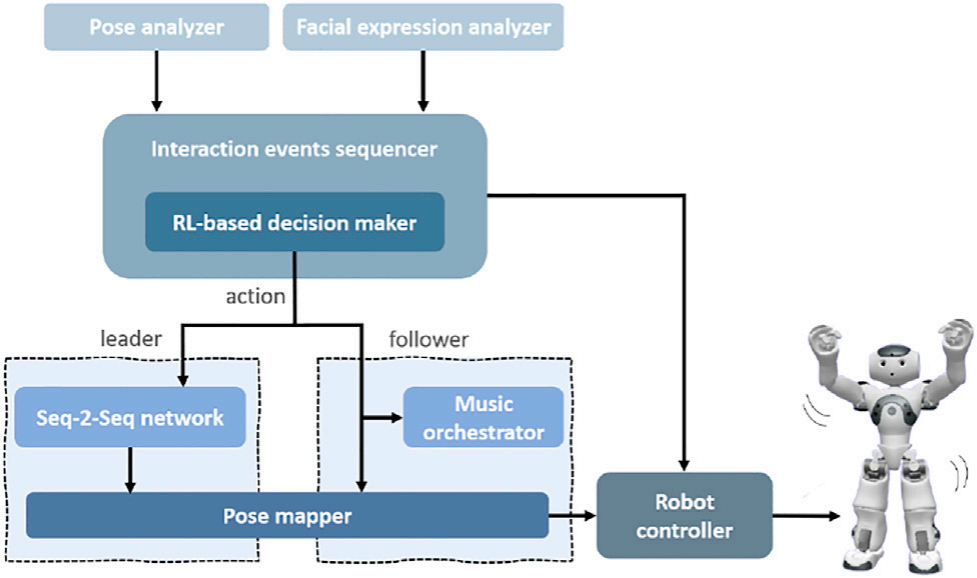
The main system components in our dance interaction design. These consist of the pose and facial expression analyzers that process behavioral cues from the user. The interaction events sequencer executes all the steps in our interaction design, within which the RL-based decision maker uses the behavioral data to determines the best robot action. The S2S network generates the robot dance sequences in the leader mode and the observed user pose data is used directly to create the follower mode robot movements and musical output. The pose mapper creates a mapping of the human poses for the robot body and the robot controller communicates all the received commands to the robot.

***System Overview*** Our robot-assisted dance-based interaction system consists of several important components. The multimodal behavioral sensing comprises a facial expression analyzer that uses Affectiva’s Affdex software (McDuff et al., 2016) and a 3-dimensional human pose analyzer that uses the Microsoft Kinect (v1) (Zhang, 2012) (or OpenPose (Cao et al., 2019) for remote data collection). It also includes a sequencer for all interaction events which contains an RL-based decision-making component that determines the most suitable next action in order to personalize the robot behavior for each user. In addition, all the commands intended for the robot are sent through a central controller module that handles all the communication with the robot. The leader role dances are generated by a trained sequence-to-sequence (S2S) (Sutskever et al., 2014) network that is implemented as an individual module. The follower role music is produced by a music orchestrator (Sandygulova et al., 2019) that generates music in correlation with the user’s poses. And lastly, we implemented a pose mapping module that realizes the dance poses in both roles onto the robot in real-time. Figure 2 illustrates these system components.

The facial expression analyzer was implemented in C#, the pose analyzer for Kinect was written in C++, the music orchestrator was written in Java, and all other components were implemented in Python, with TCP/IP connectivity to facilitate communication between the various components.

**Interaction design** There are several mechanisms that we incorporated into our dance-based interaction with a robot. Some of these were based on the techniques and concepts that are implemented in autism therapy such as prompt fading, preferred prompt types, reinforcements, and time delay. Other mechanisms discussed here were included to facilitate the interaction and/or encourage engagement, such as equipping the robot with the ability to socialize with the child and ensuring that the child remained in control of terminating the interaction.

### 2.1 Prompt Hierarchy

Stimulus prompts are supportive antecedent strategies used to encourage learning, promote independence, and nurture confidence in learners. Prompt fading (Cengher et al., 2018) is a strategy where, once the learner becomes familiar with the target, the prompting is systematically faded until the learner can produce the correct response without any external stimulus.

We used this as an inspiration to create a prompt hierarchy for our robot-mediated interaction, which includes 3 levels:

• Verbal (level 1): These include direct instructions to perform dances or increase the energy in the performance, or even general encouragement to improve the performance, but include no gestures. Examples: 1) “Come on! I know you can do better than that!” and 2) “Now can you show me your coolest dance moves?"
• Verbal and gestural combined (level 2): The robot delivers the same verbal prompts but is accompanied by gestures to emphasize the verbal instructions. These are simple gestures such as a brief dance sequence (like a shimmy), a thumbs up, a fist pump, etc. to support the verbal instructions.

Incentive (level 3): This level is included to improve the child’s attention span by providing a non-task break. There is evidence to support the use of breaks and rest during learning tasks to improve task performance (Toppino et al., 1991; Ariga and Lleras, 2011). This led us to include children’s jokes within this prompt category to provide an opportunity for the child to relax and refocus on the task at hand. The jokes are accompanied by meaningful, relevant gestures and the punchlines are followed by the playback of a laugh track. Examples: 1) “What is brown, hairy, and wears sunglasses? A coconut on vacation.” and 2) “What do you call a dinosaur that is sleeping? A dino-snore!"

The prompt fading strategy was implemented across interaction sessions as well. This means that if, for example, a child produced a desirable behavior upon receiving a level 3 prompt (incentive) for robot action A in interaction session 1, if the child requires a prompt for action A in session 2, the prompting will begin at level 2 (verbal + gestural), hence fading prompts across sessions as well as within sessions to promote independence.

### 2.2 Reinforcement Hierarchy

Reinforcement is a reward or a stimulus that follows a behavior that makes the behavior more likely to occur in the future. In line with the prompt hierarchy, we created a hierarchy of social reinforcers as a means to give positive attention to a child upon displaying a desirable behavior (Kamana, 2012). The purpose, once again, is to reduce the dependence on prompts such that unprompted positive behaviors are rewarded more than those that require prompting.

Our reinforcement hierarchy has 4 levels that progressively increase in the intensity of positive attention.

• Verbal (level 1): These are simple verbal encouragements to reinforce the positive behavior. Example: “Good job! You did it!ˮ
• Verbal and gestural combined (level 2): These include verbal reinforcers that are slightly more positive than those used in level 1, and are also accompanied by simple, positive gestures to support the verbal encouragement. Example: “That was good! You're a great dancer!ˮ accompanied with a thumbs-up gesture.
• Verbal with a celebratory dance and applause track (level 4): These include verbal reinforcers at the same level of positivity as in level 3, alongside a celebratory dance, as well as a brief applause soundtrack played in the background to form the highest level of reinforcement that the robot can provide. Example: “Bravo! I totally love your dance moves! Will you teach me too? I would love to dance like you!” accompanied by a short dance celebration.

The reinforcement levels are inversely related to the prompt levels; if a positive behavior is elicited without a prompt from the robot, level 4 reinforcement is given, but if a positive behavior is observed after delivering a level 3 prompt, only a level 1 reinforcement is provided.

### 2.3 Time Delay

Time delay refers to the amount of time between giving an initial instruction to a child and providing a prompt to help them follow the instruction (Neitzel and Wolery, 2009). When a child is first learning a new skill, the instruction and the prompt may be given simultaneously, for example, the instructor may ask a child to pick up their books while also pointing at the books. However, as the child becomes more adept at understanding the instruction, the time delay between the instruction and the prompt may be increased, subsequently helping the child complete the task more independently. These strategies are employed until the child no longer needs any prompts to execute the task or skill.

In our interaction, we implemented a constant time delay of 10 s between the instruction and the first prompt, as well as between all the following prompts in the hierarchy. This provides an ample amount of time for the music playback and the accompanying robot dance, during which all child behaviors are monitored to evaluate the response.

### 2.4 Termination Control

Autistic children are known to perform better in environments that are structured and predictable (Ferrara and Hill, 1980; Schadenberg, 2019), showing improvements in responsiveness to environmental stimuli when they occur consistently and predictably. The child-robot dance interaction presented in this work is a turn-based activity that concludes each turn by asking the user if they would like to continue participating in the interaction with the robot. This feature was implemented specifically as a measure for decreasing unpredictability, thereby minimizing possible sources of anxiety and, hence, increasing compliance. Additionally, by consistently offering this choice to the child at the end of every turn, not only are they able to maintain control over their participation, but they also learn to make decisions and think for themselves, thereby improving their self-esteem (Maxwell, 1989).

### 2.5 Robot Socialization Techniques

The social behavior of the robot is crucial in achieving a naturalistic and engaging cHRI. We incorporated several techniques to improve the sociability of the robot.

Firstly, the robot introduces itself at the start of the interaction, says hello to the child (addressing them by their name), and continues to describe the dance activity. This is done in a very friendly manner, with the robot showing excitement and eagerness to play with the child and is implemented through a combination of speech and supportive gestures.

The interaction subsequently follows a sequence of communications between the child and the robot, which can include directions given to the child for each turn and what they can expect, as well as any necessary prompts, reinforcements, and indications of changes in robot roles.

Additionally, certain interaction events may also trigger an inquiry presented by the robot to the child regarding their willingness to continue participation in the interaction. This can happen in two scenarios: 1) If the child shows a lack of interest in the activity during a turn and removes themselves from the interaction, the robot is not able to collect any facial expression or pose data, which serves as an indicator of disengagement, and 2) When the turn comes to an end. In both cases, the robot presents an inquiry to the child, asking to confirm their willingness or lack thereof to continue the interaction. Eventually, if the child wishes to conclude the interaction, a goodbye routine is triggered, in which the robot expresses excitement and appreciates the child for participating, before saying goodbye.

It must be noted here that although the interaction design remains largely autonomous, it depends minimally on human input for the child’s name and their response to the inquiry after each turn.

### 2.6 Multi-Role Robot Behaviors

To ensure bidirectional learning and interactivity, we implemented two different robot roles to enable our robot to both initiate dances in the leader role and follow the child’s dances in the follower role.

#### 2.6.1 Robot as a Follower

The first role of our robot is that of a follower. In this role, the child initiates the dances, and the robot imitates their movements in real-time. This imitation includes upper body movements and body rotations. In this scenario, the child’s body movements trigger the musical notes, such that both the angles and the velocities of the detected skeletal joints dictate the musical pattern that is generated. The changes in joint angles and velocities are continuously tracked and used to produce music that is responsive to the child’s movements. Each turn in this role lasts at least 50 s or more in case prompting is required.

*Why Follower Role?* The follower role is important in that it provides the child an opportunity to take charge of the interaction, by controlling both the music orchestration as well as the movements of the robot. This also offers an opportunity for the child to explore their own poses, movement patterns and speed, as well as their impact on the interaction dynamics. Hence, this role is designed to emphasize the skills of self-initiation and movement exploration in the child.

*Music Orchestration* This is implemented using the JFugue 4.0 library in Java (Koelle, 2016). The pose analyzer captures and tracks the changing full-body poses of the child, which, in the follower mode, are sent to the music orchestrator (Ryan et al., 2018) in real-time. Several conditions are preset and stored in the music orchestrator that defines a mapping between joint angle and velocity ranges and the musical notes, which are played when the values received from the pose analyzer fall within the said ranges.

Our conditions included the angular and velocity data points for both left and right shoulders, elbows, hands, knees, and feet. The angular conditions were defined by splitting the entire range of motion (as detected by Kinect) for each joint into two ranges for the legs and six ranges for the arms, while the velocity conditions included two ranges to indicate slow and fast movements for both arms and legs.

**FIGURE 3:**
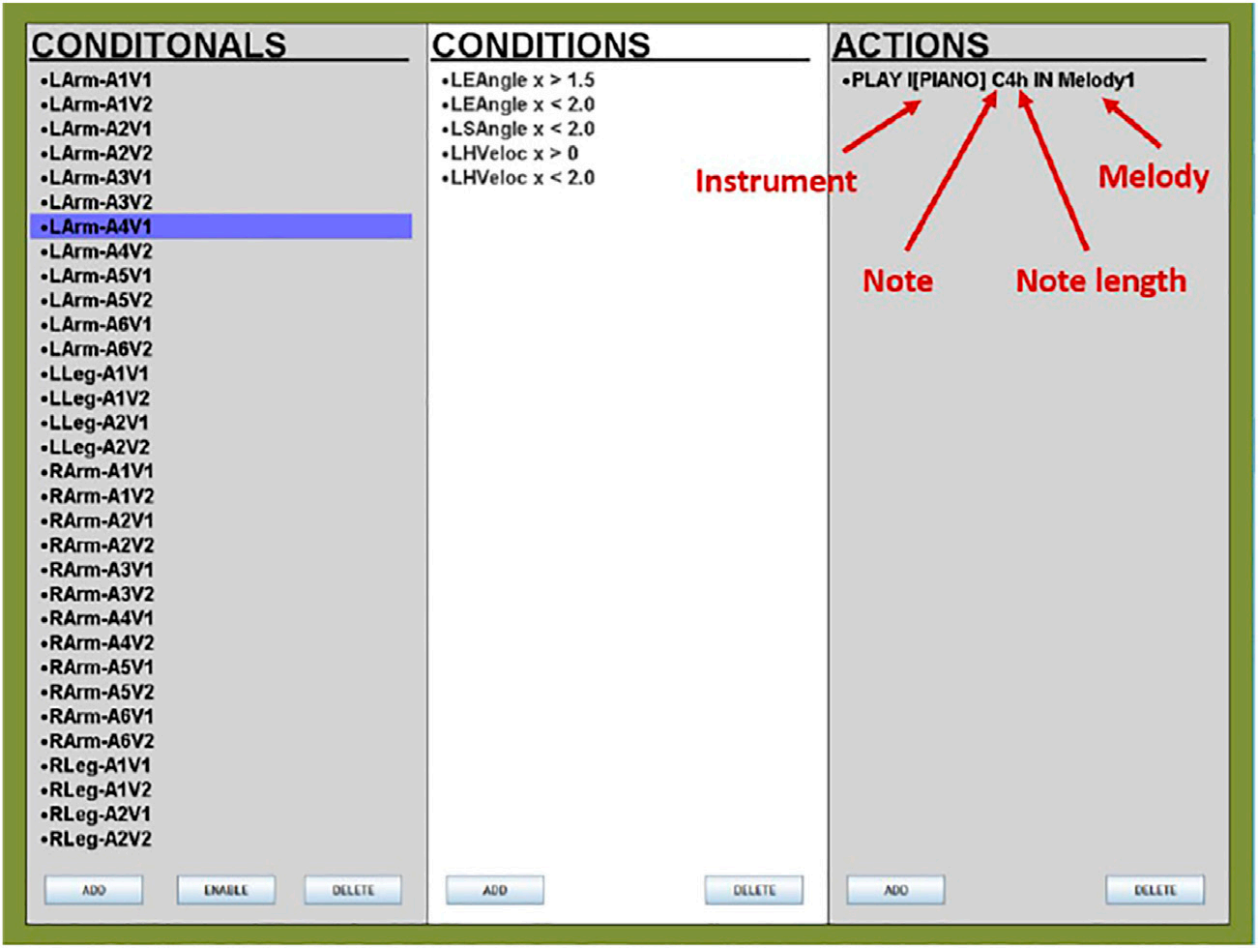
The graphical user interface we used to define the pose-to-music mappings in the music orchestrator (Alankus et al., 2005). A breakdown of the action command that generates the music output is also shown, highlighting the instrument, note, note length, and melody settings.

The GUI used to facilitate the design of these conditions is shown in Figure 3. The column on the left shows the names of each conditional, the middle column describes the conditions set within each conditional, and the action shows the details of the musical output when the conditions become true. Within the action, the musical instrument, as well as the length and melody of the musical note, are defined, as highlighted in the figure. Any conditional can easily be enabled or disabled as required: for example, if, for any reason, a child insists on being seated during the interaction, we can disable all the left and right leg conditionals from the GUI so that all musical output is determined only by the upper body movements.

We mapped each of the four limbs to a different musical instrument so that a variety of sounds are generated. A total of 128 different instruments and sound effects are available in the JFugue sound bank, including several different types of guitars, pianos, and pipe and string instruments, in addition to some ethnic instruments and other miscellaneous sound effects. The four instruments used for our mapping were the piano, electric bass, electric clean guitar, and the slap bass. The use of the JFugue streaming player allows the dynamic generation of musical patterns at runtime, bypassing the need to construct the entire pattern before it can be played.

Therefore, the aforementioned methods enable the child’s body to act as a musical instrument by making changes in body pose responsible for the musical output within this robot role.

#### 2.6.2 Robot as a Leader

The second role of our robot is that of a leader in which the robot initiates the dancing. The dances are performed to popular children’s songs that are anticipated to be familiar to most children. These are upbeat, high-energy songs that are played in the background at each turn within the leader role. The songs are sampled randomly in every leader role turn from a library of 15 songs without replacement. The children are expected to dance along with the robot but are free to mimic the robot’s movements or to create their own. The robot dances are choreographed through the trained S2S network that produces the dance sequences for the selected song at runtime, which are then realized on the robot through the pose mapper. Each turn in this role lasts at least 50 s or more in case prompting is required.

***Why Leader Role?*** The leader role is designed to provide the child an opportunity for physical activity and imitation. The high-energy music aims to encourage movement, which may include dance moves that the child may already know from previous exposure to the songs. It also serves to practice imitation skills, where a child may mirror the poses being shown by the robot.

***Sequence-To-Sequence Learning for Dance Synthesis*** As mentioned previously, the lack of effectiveness of existing robot-mediated dance interactions for autism can be partly attributed to the limited dancing abilities of the robot. We wanted to enable our robot dancer to perform to a variety of different songs, without committing to the labor of manual choreography design. This led us to formulate this as a sequence-to-sequence prediction problem that uses a Long Short Term Memory (LSTM) encoder-decoder network to learn the mapping between the audio features of the music and the motion features of the corresponding dances. The trained network is then used to make runtime predictions of the dance sequence output for a given song, where the dance output is conditioned on the music features.

**FIGURE 4:**
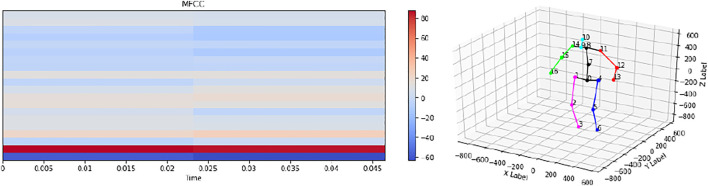
Visualizations of sample MFCC and pose inputs given to the LSTM encoder-decoder network that outputs dance sequences for the robot in the leader mode.

We used 15 YouTube videos of children’s dances to create our own database of music-to-motion feature sets. We extracted the audio features for each of the 15 songs in the database. These consisted of the Mel-frequency cepstral coefficients (MFCCs), which represent the short-term power spectrum of a sound. Additionally, we used a variant of OpenPose that generates 3D human pose estimates for real-time processing to extract movement features from the videos. The dances included in these videos are choreographed, not freestyled, meaning that we were able to extract multiple instances of the same dance sequence from a single video. Figure 4 shows the visualizations of sample inputs to the network, i.e., the MFCCs and the motion data. We used simple, single LSTM-layered encoder-decoder models used for the training and inference processes, where we formulated the music-to-dance synthesis as an MFCCs-to-joint-coordinates mapping problem.

For training, we used a learning rate of 0.0005 with a decay rate of 0.001, included the RMSprop (Root Mean Square Propagation) optimizer for performance improvements, and used mean squared error (MSE) as the loss function. An 80:20 training to validation split was used in the training process for 100 epochs, with a batch size of 8. The training process resulted in an MSE value of 0.0018.

The primary purpose of this approach was to achieve automated choreography synthesis for the dancing robot. Though the resulting dance sequences can be improved in terms of the meaningfulness of expression and synchronization to the music beat, a simple approach such as the one presented here was sufficient for our purpose. Other research efforts have utilized more sophisticated audio processing and deep learning techniques to improve the quality of the dance output (Zhuang et al., 2020; Ye et al., 2020; Tang et al., 2018), but these have mainly been applied to characters and avatars that do not necessarily present the same challenges as embodied robots.

### 2.7 Social Perception: Modeling and Interpreting Behavioral Cues

Our user engagement model was designed to reflect both **affective engagement** and **task-related engagement**. This was done to encompass the affective responses that are exhibited through behavioral cues such as facial expressions and eye gaze attention, which can sometimes manifest subconsciously, as well as physical responses to direct dance requests or prompts from the robot. This multimodal representation was chosen to capture a comprehensive measure of child engagement in the interaction. The modalities were also carefully chosen to be suitable for remote data collection entirely through a live video stream in virtual settings.

Affective engagement has two components: **emotional engagement** and **attentional engagement**. Emotional engagement is measured by the emotions expressed through facial expressions, and attentional engagement is measured by the eye gaze attention given to the robot during the interaction. Therefore, positive emotional expressions with the gaze focused on the robot would yield higher affective engagement than neutral or negative facial expressions with the face turned away from the robot. The emotional engagement was categorized into 3 states: negative, neutral, and positive. The attentional engagement was also categorized into 3 states: unengaged, semi-engaged, and fully engaged. Since Affdex assigns a confidence value to each output feature, we used these values to create an empirical mapping from its output values to our states. For the emotion state mapping, joy was mapped to the positive state, all other emotions were mapped to the negative state, and valence was used to determine when these confidence values were in a range that was low enough to qualify as a neutral state. A surprise was considered to be a negative emotion based on the findings from (Noordewier and Breugelmans, 2013). For attentional engagement, empirical threshold values were used to define the mapping between the attention coefficient and the attentional states that we defined for our model. Details of these mappings are shown in Table 1.

**TABLE 1:** A summary of the mappings between the detected behavioral features and the state values we defined for our model.

Data Source	Feature	Observation	Metric	State Mapping
Affdex	Joy, Disgust, Contempt, Fear, Anger, Surprise, Sadness, Valence	**Emotional engagement states**	Max average coefficient over response period	Max average coefficients belongs to + emotion → **positive**
				Max average coefficients belongs to—emotion → **negative**
				If + and—max average emotion coefficients are equal → **positive**
				If (max average coefficient in low range (<0.2) AND -15 < valence < +15) → **neutral**
Affdex	Attention	**Attentional engagement states**	Average coefficient over response period	<30 → **unengaged**
				30–80 → **semi**-**engaged**
				>80 → **fully**-**engaged**
Kinect OR OpenPose	Angular joint velocities	**Task engagement states**	$\sqrt{\sum_{\text{i}}\left({\text{v}}_{\text{i}}^{2}\right)}$ where i is the joint number	Average metric over response period >2 → **high**
				Average metric over response period ≤ 2
				• if the number of peaks sustained over 4 s during 50-s observation duration≥ 4 → **high**
				• if the number of peaks sustained over 4 s during 50-s observation duration <4 → **low**
				• if the number of peaks sustained over 4 s during 10-s post-prompt observation duration ≥ 1 → **high**
				• if the number of peaks sustained over 4 s during 10-s post-prompt observation duration <1 → **low** where a peak is detected when the average metric over a 4-s interval >2

In the context of the dance interaction presented here, **task-related user engagement** is measured by the physical activity level of a child when leading or following the robot in a dance. This was quantified through the Euclidean norm of the angular velocities of all the tracked skeletal joins available from the pose data. This physical activity level was then classified into two states: high or low, and an empirical threshold was set to distinguish between the two states. However, given that autistic children are often reported not to be as physically active as their TD counterparts, we did not consider it feasible to expect a child to sustain a high level of physical activity throughout the duration of a turn. Therefore, we introduced the concept of activity peaks. A high physical response maintained over a duration of 4 s was considered a peak. If the average activity level measured during an observation period was above the set threshold, the activity level was considered to be high. On the other hand, if it did not meet the threshold, we measured the number of peaks. If four peaks were found in the 50-s observation period or one peak in the 10-s post-prompt observation period, the detected activity level was considered to be high. Otherwise, it was mapped to the low activity state. Details can be found in Table 1.

To quantify the engagement states, we first mapped our component states to numerical values:

• Emotion states: {positive, neutral, negative} → {1, 0.5, 0}
• Attention states: {fully-engaged, semi-engaged, unengaged} → {1, 0.5, 0}
• Task engagement states: {high, low} → {1, 0}

Overall engagement, *eng*, is then measured as:

$$eng=eng_{aff}+\;eng_{task}$$ (1)

where *eng*_*aff*_ is the affective engagement and *eng*_*task*_ is the task engagement. In addition, *eng*_*aff*_ is computed as:

$$eng_{aff}=eng_{emo}+\;eng_{att}$$ (2)

where *eng*_*emo*_ is the emotional engagement and *eng*_*att*_ is the attentional engagement. By these definitions, 0 *≤ eng*_*aff*_ *≤ 2* and 0 *≤ eng ≤ 3*.

### 2.8 Platform for Virtual Data Collection and Its Limitations

Due to the outbreak of the Coronavirus pandemic at the time this research was being conducted, we were forced to conduct our user study in a remote setting, allowing children to participate in this work from the safety and comforts of their own homes. The study was performed over video meetings conducted through video conferencing applications, which meant that all the required behavioral data needed to be acquired solely from the video stream. This implied that we could no longer use the Kinect sensor to collect pose data and had to switch to using the OpenPose variant to generate the 3D pose estimates in real-time. Since the quality and accuracy of the pose data from OpenPose are lower than that from Kinect, our sensing techniques were compromised to some extent, but this was considered a necessary measure under the circumstances.

In addition to this was the poor picture quality resulting from network-related issues, which is a bigger problem in that our only data source can potentially be compromised or frequently disrupted over the course of the interaction. There were additional challenges of working with minimal control in remote settings: the rooms from where the children often participated in these sessions were often not well-lit, did not offer enough space to capture full-body pose data, and had many distractions in their backgrounds that contributed to the difficulty of capturing accurate 3D pose estimations. The data presented in the user study were, thus, collected through our virtual experiment framework. Instead of using the real robot for this setup, we chose to utilize a simulation of Nao, which was shown to the participating children through screen-sharing during the virtual interaction sessions.

#### 2.8.1 Experimental Procedure and Setup

We disclosed the interaction procedure and research purpose to the parents of the participants prior to conducting the study. We also obtained consent from the parents for their children’s participation in our user study. The children participated in two sessions, where they interacted virtually with the robot through video meetings. The parents and the children were both informed that they could terminate a session at any time without consequence. As a result, the sessions were of variable lengths, dependent upon the child’s interest and willingness to continue. The first session formed the pretest condition and the second formed the post-test condition.

The children participated in the study from their homes. The parents typically set up a computer with a webcam in a dedicated room, but the experimental settings remained largely uncontrolled. The children were asked to remain standing in front of the computer, at a distance that allowed a full-body view to be captured by the camera, but once again, this condition was not strictly controlled. Despite these limitations, we were able to derive some interesting findings from the experiments conducted through our virtual setup.

#### 2.8.2 Measures

Given the small size of this study, we employed descriptive statistics to evaluate the pretest and post-test conditions in this study. These include an assessment of the:

1) **General interaction metrics**: To identify any changes in the children’s behaviors as a result of the robot role, we compute the total number of turns, number of turns in the leader role, number of turns in the follower role, total number of prompts used, number of prompts used in the leader mode, number of prompts used in the follower mode, and number of successful turns (i.e. number of turns eliciting a high level of physical activity). These metrics also help to draw a comparison between the engagement generated by the two types of robot roles.
2) **Impact of mixed robot roles**: We define engagement gain as the difference in child engagement levels between the pretest and posttest conditions, which assesses any changes in the engagement from the single-role to the mixed-role mode. Measuring engagement can be complex given the highly individualized nature of the expression of interest. In an effort to obtain a metric that is standardized across the participants, we use the normalized differences in the pretest and post-test conditions to obtain this metric. We define normalized change, *c*, as:

$$c=\{\begin{array}{c} \frac{eng_{post}-eng_{pre}}{eng_{max}-\;eng_{pre}};if\;eng_{post}>eng_{pre}\\ drop;\;if\;eng_{post}=eng_{pre}=eng_{max}\;or\;0\\ 0;\;if\;0<eng_{post}=eng_{pre}<eng_{max}\\ \frac{eng_{post}-eng_{pre}}{eng_{post}};if\;eng_{post}<eng_{pre} \end{array}$$ (3)

where *eng*_*pre*_ is the average engagement level in the pretest condition and *eng*_*post*_ is the average engagement level in the post-test condition, and *eng*_*max*_ is the maximum value *eng*, which equals 3 based on Eqs 1 and 2. Additionally, we also compute the changes in the raw values of the individual components of the overall engagement metric, which are emotion, attention, and physical activity levels.

#### 2.8.3 Participants

The targeted inclusion criteria for this study included children of ages 5–8 years, who have been diagnosed with autism and have verbal skills, with English being the primary language spoken at home. We were, however, able to recruit only 3 participants that did not meet all the aforementioned conditions. The demographic information is summarized in Table 2. All three participants were Asian, non-verbal and did not primarily speak English at home. Two of the 3 participants also exceeded the target age range for this study, with the mean age of the 3 participants being 9.3 years. Though the clinical details of the diagnoses of all 3 participants remained undisclosed, we were able to estimate the severity of their symptoms through a symptom checklist (CHC Resource Library, 2021) filled out by the parents. These showed that all three participants experienced a majority of the typical autism symptoms, including limited social skills, lack of social interactions, repetitive behaviors, avoiding eye contact, and lacking empathy, etc. The parents of Participants one and 3 described them as having little to no interest in music or dancing, whereas Participant two was reported to be fond of musical activities. Given the size of the study, we relied on qualitative analysis to conduct a preliminary evaluation of our system to inform a larger-scale future study.

**TABLE 2:** Demographic information for the participants of the user study.

Participant id	Gender	Age	Race
1	M	10	Asian
2	F	7	Asian
3	M	11	Asian

## 3 Results

We evaluated the single-role sessions versus the mixed-role sessions for all 3 participants to validate the effectiveness of the mixed-role design in engaging children and encouraging physical activity. This section presents the results of this evaluation.

### 3.1 General Metrics

The descriptive metrics from all the sessions are summarized in Table 3. The order of the two single-role designs was counterbalanced. The participants took part in a total of seven turns in the follower-role-only design, 13 turns in the leader-role-only design, and 23 turns in the mixed-role design. It should be noted that most of the turns in the mixed-role design are attributed to Participant 2, which is discussed later. Out of these turns, there were seven successful turns in the follower-role-only design, 12 in leader-role-only design, and 23 in the mixed-role design, where success in a turn is achieved when a child’s physical activity is measured as high. This shows that most turns were able to elicit a high physical response from the children irrespective of the interaction design.

**TABLE 3:** Summary metrics for the three participants from our user study that assesses the impact of interacting with the robot in single roles versus mixed roles on child engagement (L: Leader, F: Follower, M: Mixed).

Id	Session Sequence	Session type	Total # of turns	Total # of Successful turns	Total # of prompts	Most frequent emotion State	Most frequent attention state	Task engagement State
1	1	F	1	1	0	Negative	Semi-engaged	High
	2	L	4	3	3	Positive	Fully-engaged	High
	3	M	2	2	0	Positive	Fully-engaged	High
2	1	L	8	8	1	Positive/Negative	Fully-engaged	High
	2	F	3	3	0	Positive	Fully-engaged	High
	3	M	19	19	0	Positive	Fully-engaged	High
3	1	L	1	1	1	Neutral	Unengaged	High
	2	F	3	3	0	Positive	Fully-engaged	High
	3	M	2	2	0	Negative	Fully -engaged/Semi-engaged	High

Additionally, 0 prompts were given in the follower role only design, five in the leader-role-only design, and 0 in the mixed-role design, suggesting that the robot behavior in the follower-role and mixed-role designs were perhaps more engaging for the children, thus requiring no prompting. However, it would be misleading to accept this interpretation since it was very clear to all human observers of the child-robot interactions that the leader role design was more effective than the follower role. The reasons are discussed comprehensively in the Discussion section.

As can be seen from Table 3, the most frequently observed task engagement state, i.e., the physical activity level, was high across all 3 interaction designs. However, the most frequently observed emotion and attention states contain mixed results but cannot determine on their own if one interaction design is more or less effective than the others.

### 3.2 Impact of Mixed Robot Roles

*Paired T-test*: To compare the engagement metrics from the single-role sessions versus the multiple role sessions, we ran paired *t*-tests (∝ = 0.05) on the average overall engagement, average emotion, average attention, and average task engagement measures obtained from the 3 participants. Hence, there were two paired t-tests for each of the four measures, one to compare the performance metrics of the leader role session vs mixed-role session, and the other for the follower role vs mixed-role session. This was done to evaluate if the mixed role design had a statistically significant impact on the engagement of a child compared to either of the single-role designs. However, we found no statistically significant difference for any of the evaluated measures (*p*-value > 0.05). This is not surprising given the small sample size used in this study. However, we did observe promising trends from our framework, as discussed below.

**FIGURE 5:**
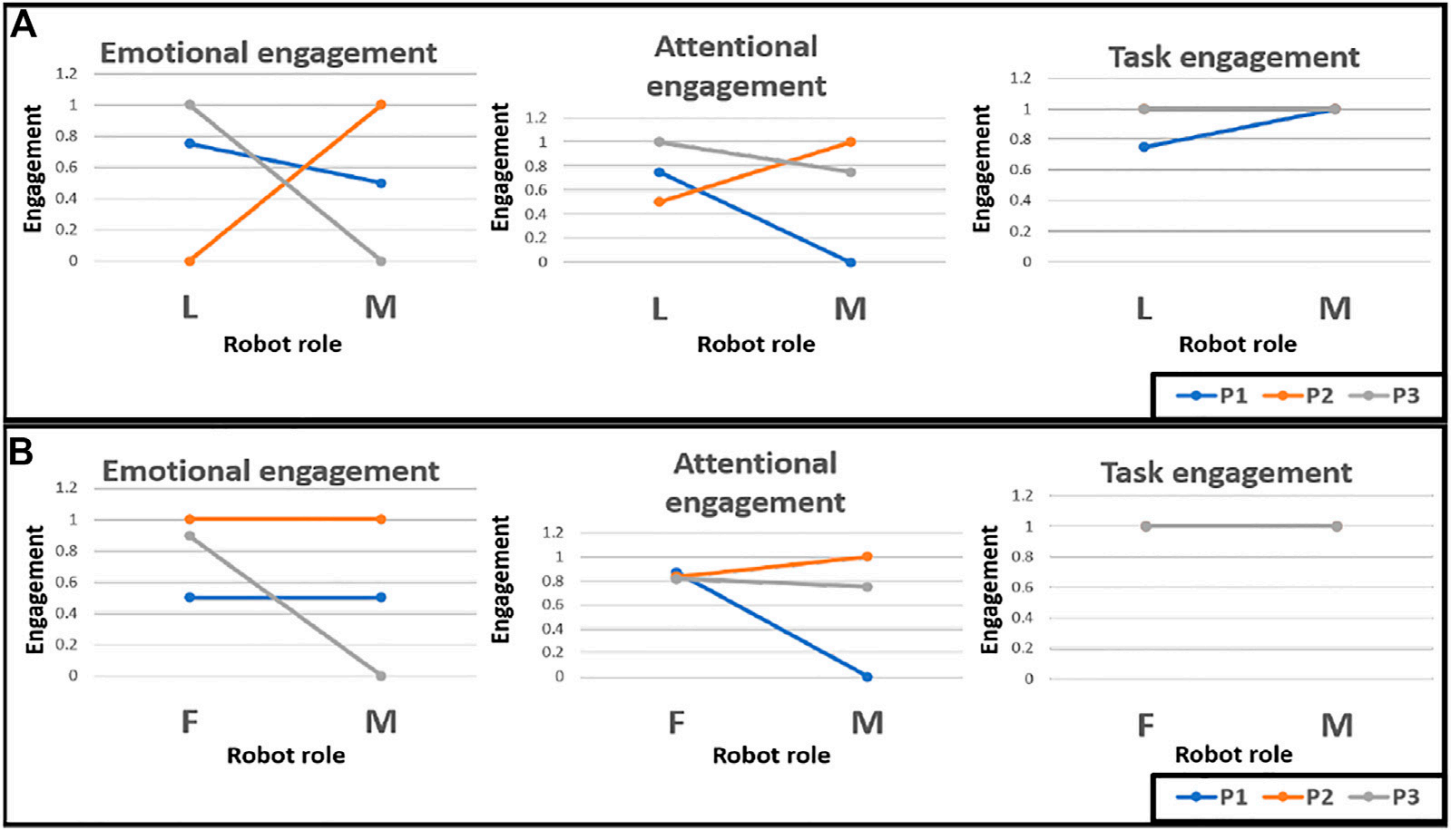
Changes in the raw values of the engagement components under the single-role and mixed-role designs, where **(A)** shows the changes between the leader role only vs mixed-role conditions, and **(B)** shows the changes between the follower role only vs mixed-role conditions. P1, P2 and P3 are the three participants from our user study (L, Leader, (F) Follower, M, Mixed).

Figure 5 shows the changes in the raw average values of the 3 measures between the single and mixed-role designs. Considering Figure 5A, which depicts the changes between the leader role only and the mixed-role designs, it can be seen that the *average* ***emotional and attentional engagement*** for Participant two *increased* in the *mixed-role interaction*, while those for the other two participants showed a decreasing trend. For ***task engagement***, participants two and 3 maintained a high state for *both interaction designs*, while Participant one showed an *increase* in task engagement for the *mixed-role interaction*.

From Figure 5B, participants one and two are seen to maintain their ***emotional states*** between the *follower-role and mixed-role designs*, whereas Participant 3 showed a decline in emotional engagement in the mixed-role interaction. For ***attentional engagement***, Participant two showed an increase, Participant 3 showed a slight decrease while Participant one showed a larger decline for the *mixed-role design*. For ***task engagement***, all three participants maintained a high physical activity level across *both interaction designs*.

Overall, we have observed the trends of 1) increased emotional, attentional, and task engagements in the follower-role mode (in which the child is the leader), 2) maintained or slightly increased emotional engagement for follower-role and mixed-role designs, and 3) increased or maintained task engagement for all interaction designs.

The raw engagement values and normalized change values for the 3 participants are listed in Table 4. These include the changes between both the pretest conditions (leader role only and follower role only) and the posttest condition (mixed roles). Based on Eq. 3, the change values indicate a gain for both conditions for Participant two and losses for Participants one and 3. A **paired t-test** was performed for the normalized change data for the 3 participants under the L vs M and the F vs M conditions but found no statistically significant change in the *c* values (*p*-value > 0.05). Although the data, especially due to the small size, does not report any statistical results, we did notice the trends of effective increase and positive outcomes with the mixed-role in Participant 2, with whome the effect lasted for the duration of the sessions. For the other participants, we did notice their differences in their preferences toward music or dance, which we believe may have played the role in the decreased engagement. The spectrum of differences and diverse responses are common so our small group size is definitely not enough to draw any conclusions. However, with our study being conducted online with many technical and societal difficulties during the COVID-19 pandemic, we believe our efforts and findings in a personalized interaction framework with adaptive prompting could bring insights to other clinical intervention studies.

**TABLE 4:** Overall engagement values and normalized engagement changes for the three participants, with the robot in a single role versus mixed roles (L: Leader, F: Follower, M: Mixed). A gain in engagement occurs only for Participant 2.

Participant	Overall engagement*, eng*			Normalized change, *c*	
	*eng*_*pre*_ (L)	*eng*_*pre*_ (F)	*eng*_*post*_ (M)	*eng*_*pre*_ (L) vs *eng*_*post*_ (M)	*eng*_*pre*_ (F) vs *eng*_*post*_ (M)
1	2.250	2.375	1.500	−0.500	−0.583
2	1.500	2.834	3.000	1.000	1.000
3	3.000	2.711	1.750	−0.7143	−0.549

## 4 Discussion

The 3 participants interacted with our dancing robot in 3 different sessions where the robot operated 1) only in the leader role, 2) only in the follower role, or 3) in mixed roles. One and two are the pretest conditions and 3 is the post-test condition in this study. While the size of the study prevented us from deriving any definitive conclusions about our hypothesis, i.e., the superior engagement capability of our mixed-role design over the single-role designs, we made some very interesting observations to inform the future of this work.

Firstly, the nature of the two robot roles is very different: contrary to the follower role behaviors, robot dances in the leader role is determined by the output of a pretrained S2S network with no dependency on the real-time behavioral data tracked during the child-robot interaction. Robot dances in the follower role are a real-time imitation of the child poses captured during our video-based virtual data collection process. Therefore, any noise or disruption in the video directly impacts the follower role output, i.e., the robot movements. This was obvious in this study, not only because the video quality was often compromised during the interactions, but also because the video-based, real-time 3D pose extraction naturally underperforms compared to a sensor such as Kinect. It also had implications for the real-time measurement of the children’s behavioral responses (emotion, attention, and physical activity), the very source of which is the video stream.

This was clearly reflected in the children’s overarching response to the two types of robot dances as well. The leader role dances were driven by the music that was already familiar and fun for the children. The follower role dances were driven by the movements and music orchestration that were both dependent on the real-time capture of behavioral data. While the leader role was a clear favorite for all 3 children, we do not necessarily expect this to be the case in an in-person study in the future. We anticipate that imitations and music generation in the follower role will be far more accurate, fluid, and responsive when the data source is less noisy, making it easier for the children to perceive that they control the robot and the music in this mode, which, in fact, is the very motivation for the follower role design.

The observed trends in increased emotional, attentional, and task engagements in the follower-role mode, as explained in the Results section, aligns with the above analysis. It also resonates our intention and rationale to design more adaptive and interactive robotic framework to provide increased and natural engagements during interventions, where we believe that the goal is to nurture more motivation and active minds to the child through cHRI.

As a result of these factors, the metrics and trends shown in Table 3, Figure 5 cannot be seen as conclusive but serve well to inform the design of future research. Additionally, it must also be noted that any trends based on quantitative metrics for this exploratory study are expected to vary considerably as the dataset expands.

It is also important to consider a breakdown of the robot role transitioning within the mixed-role interactions. Though the details of its implementation are excluded from this paper, it is important to mention that the transitions were determined by a reinforcement learning-based strategy. RL algorithms are known to be data-hungry and require many data points to reach convergence in the training process. As a result, in the 3 mixed-role interactions that are included in this study, the follower role dominated the robot behavior. The interactions for participants one and 3 did not last long enough for them to experience a truly mixed-role design (2 turns each in follower role only), but this was not the case for Participant 2, whose session continued for 19 turns (13 turns in follower role, six turns in leader role), providing the algorithm sufficient data to start exploring the action space.

It would also be useful to evaluate the results in the context of the behavioral tendencies of the 3 participants. Participant 2, as gathered from the parental reports, was clearly the most engaged of the 3 children. She had a hearing impairment which prevented her from fully understanding the robot’s instructions, but since she was very fond of music and dancing. Moreover, since her mother played an active role during her sessions to keep her focused, she was the participant that showed the most enthusiasm for her sessions and participated for the longest durations, as reflected in Table 3. While the other two participants both seemed more comfortable in the second session with the robot (as well as the human experimenters whose presence may have added to any possible social anxiety), their sessions remained significantly shorter than those of Participant 1.

The above findings also shed light on the results presented in Table 4, where only Participant two shows a gain in engagement while the other two participants show losses. These can be attributed to both the higher initial interest level of Participant two in music and dancing, as well as their prolonged interaction with the robot enabling the personalization strategy to take effect.

Our current work does have limitations, including the small subject group size due to the restrictions during the COVID-19 pandemic, limited physical behavioral representations made by the robotic platform, especially due to the virtual characteristics of the platform, and the duration of the sessions not being long enough to collect large data to sufficiently train our reinforcement algorithm. However, with the current limitations, we believe our results do present a contribution to the field, especially of timely value to advance clinical and interventional studies to cope with pandemics and telehealth.

**FIGURE 6:**
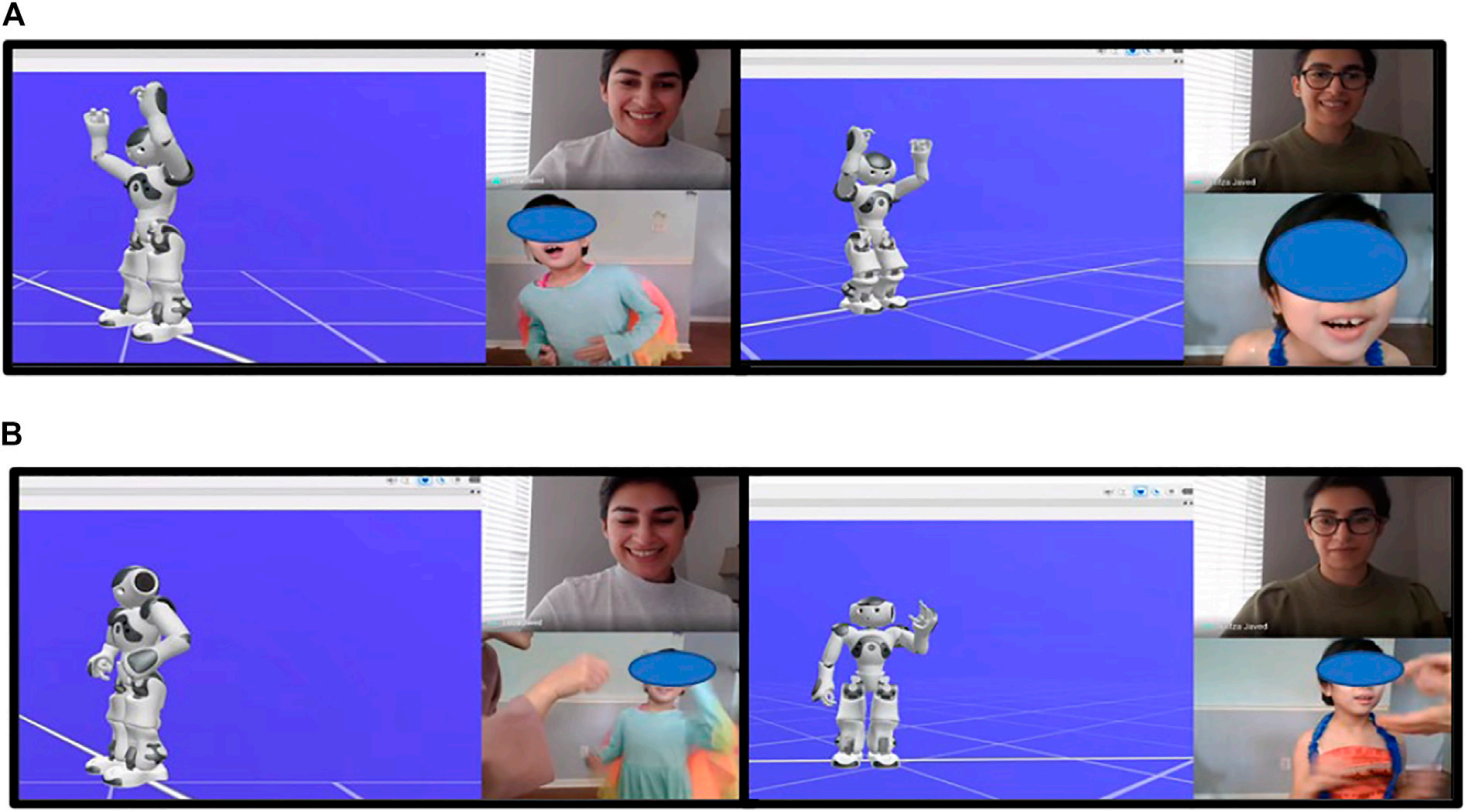
Scenes depicting some important interaction events from our user study to evaluate the single-role versus mixed-role designs. **(A)** Participant shows fascination with the robot celebrations, and **(B)** Parent uses the end of a turn to encourage verbal + signing skills in the participant (Written informed consent was obtained from the individual(s) AND/OR minor(s)' legal guardian.).

A note must also be made of the importance of the parent’s involvement in the virtual interactions. Participant 2’s mother was very actively involved in her interactions with the robot and found creative ways to use the setting to encourage her daughter to practice verbal/language skills as well. She would ensure that at the end of every turn, the interaction would not proceed until the participant vocalized her responses. These responses included vocabulary such as “more,” “yes,” “no,” “more music,” “more dancing,” “stop” and “keep going”. Examples of such incidents are shown in Figure 6A. The interaction design was, therefore, used to motivate speech in the participant as well, and can possibly be leveraged in the future to elicit other desirable behaviors.

Another interesting observation was the children’s responses to the robot reinforcement behaviors, particularly level 3 and level 4 reinforcers that utilized celebratory dancing and/or applause in the background. These were very highly effective in that their interpretation was very clear and did not require any language skills. The participants were observed to respond to these celebrations with smiles, laughs, and even imitative behaviors to mimic the robot gestures. Some scenes depicting these incidents are captured in Figure 6B.

## 5 Conclusion

We implemented a robot-assisted dance interaction system as a means to promote physical activity in autistic children through the use of music- and dance-based interactions. We also incorporated the largely unexplored paradigm of learning-by-teaching by incorporating multiple robot roles that allow the child to both learn from and teach to the robot. Our work is among the first to implement a largely autonomous, real-time full-body dance interaction with a bipedal humanoid robot that also explores the impact of the robot roles on child engagement. We conducted an exploratory study to evaluate the effectiveness of our design and found no statistically significant difference in the children’s engagement in single-role and multiple-role interactions. While the children were observed to respond positively to both robot behaviors, they preferred the music-driven leader role over the movement-driven follower role, a result that can partly be attributed to the virtual nature of the study.

Our study made some interesting findings regarding the role of the parent during the child-robot interaction. While a parent’s presence can help children feel more comfortable, their creative involvement can help children practice skills that are not directly targeted by our interaction design, such as language skills. Since parents understand the unique needs of their children more than others, they can use our platform in creative ways to target the elicitation of specific desirable behaviors. Our work also supports the use of therapy constructs such as reinforcements, which serve to reinforce positive behaviors, are entertaining for the children, and can be useful in eliciting imitation skills in children. An in-person user study in the future will help fully exploit the benefits of an embodied interaction with a robot rather than a virtual character.

To conclude, the purpose of this study was to design a reinforcement framework to provide more adaptive and autonomous social interaction framework and report the pilot study with observed trends of our novel approach. We have observed the trends of increased emotional, attentional, and task engagements with our proposed follower-role interaction design, in which the child is the leader with the robot taking the follower role, and all participants showed increased task engagements in our cHRI sessions while each child showed different positive responses to each interaction design. We believe our approach of automated and personalized role switching with adaptive prompting can contribute to further developments in personalized medicine and clinical interventions with assistive robotic systems. Future studies will include comparison studies with typically developing children, with more advanced reinforcement learning algorithms and deep learning frameworks, and clinical evaluations in long term with our clinical collaborators. Long-term interaction and longitudinal analysis (Clabaugh et al., 2018; Clabaugh et al., 2019; van Otterdijk et al., 2020) are very important topics in HRI, especially for the robots to function more effectively “in-the-wild” (Park et al., 2020), and our future efforts plan to address these factors more dedication and collaboration.

## Data Availability

The raw data supporting the conclusion of this article will be made available by the authors, after proper de-identification adhering to our IRB protocol.
